# Trait-mediated diversification in nematode predator–prey systems

**DOI:** 10.1002/ece3.36

**Published:** 2011-11

**Authors:** Christian Mulder, Johannes Helder, Mariëtte T W Vervoort, J Arie Vonk

**Affiliations:** 1 National Institute for Public Health and Environment (RIVM)Bilthoven, The Netherlands; 2 Laboratory of Nematology, Wageningen University (WUR)Wageningen, The Netherlands

**Keywords:** Adults and juveniles, body size ratios, nematode behavioural ecology, predator–prey relationships, soil food webs

## Abstract

Nematodes are presumably the most numerous Metazoans in terrestrial habitats. They are represented at all trophic levels and are known to respond to nutrient limitation, prey availability, and microbial resources. Predatory nematodes reside at the highest trophic level, and as such their feeding habits could have a major impact on soil food web functioning. Here, we investigate the effects of gender and developmental stage on the nematode body sizes in coarse and loamy soils. Besides Neodiplogasteridae, our predators are much larger than other soil-dwelling nematodes from their early developmental stage onwards. From juvenile to adult, the predatory *Aporcelaimellus* (Kruskal–Wallis *P* < 0.001), *Dorylaimoides,* and *Tripyla* (both *P* < 0.01) show great length increases during their developmental growth, in contrast to their possible prey (almost all *P* < 0.001). Less than 4% of the prey exceeds the length of the predatory adults, but more than 30% of the prey exceeds the length of the predatory juveniles. Potential body size ratios and some physical problems experienced by small fluid feeders attacking large prey are discussed in an attempt to summarize different prey-searching mechanisms and aggregative predatory responses in the soil system.

## Introduction

Demographic consequences of endogenous traits such as body size (e.g., Amarillo–Suárez et al. 2011, and references therein) are known. Under controlled conditions, for instance, the expected initial advantage of large-sized invertebrates is affected by resource quality and increasing competition already at early life stages (Amarillo–Suárez et al. 2011). In terrestrial biota, controversy exists over whether demographic responses reported from experimental conditions might reflect ecosystem functioning (Waide et al. 1999; Hooper et al. 2005; Crutsinger et al. 2006, etc.) and environmental conditions (Crutsinger et al. 2006; Mulder and Elser 2009).

Trait variability of soil nematodes according to their gender and life stage is expected to affect the susceptibility to predation, but complex interactions between free-living nematodes of different lengths and changing soil properties are still hardly known and belowground sex-selective predation is neglected. At least in soil nematodes, gender is likely to be a key factor affecting trait variability. Sexual dimorphism in size (length) makes female nematodes larger (longer) than male nematodes (Goodey 1963; Yeates 1967, 1987; Bongers 1988; Yeates and Boag 2003), a mechanism that could make females a more rewarding target.

We anticipate that in predator–prey systems, most of the arising intraspecific trait variability reflects the diet and the feeding behavior of predatory adults. Using mathematical evidence derived from empirical studies (Mulder and Elser 2009; Mulder and Vonk 2011), we describe the extent to which life stage and gender might influence the feeding strategy of predatory nematodes.

For this purpose, we document here the body size ratios for 148,063 predator–prey links as derived at individual level (body size as length in µm) from one matrix established for every possible nematode target (prey). All the sites are managed grasslands spread across the Netherlands: 76 of them are on podzols and regosols (henceforth sand) and 29 are on fluvisols, cambisols, and anthrosols (clay). (One of the 76 sites on sand was removed from the analysis due to the lack of predatory nematodes in the recorded specimens.)

## Methods

For each soil system, a composite sample was obtained by mixing 320 soil cores randomly collected in the upper 10 cm (each topsoil core with a diameter of 2.3 cm), and approximately 500 g of soil was collected in glass jars and stored at 4°C (Mulder et al. 2003, 2011). Within one week, living nematodes were extracted from 100 g of wet soil using the Oostenbrink funnel elutriation method complemented with sieving and cotton–wool extraction (Oostenbrink 1960). In permanent mounts in formaldehyde, about 150 individuals were identified by light microscope at 400–600× and assigned to species (Bongers 1988) and related feeding habits (Small 1987; Yeates et al. 1993). We used these habits to generate a large number of expected “who-eats-whom” combinations without feeding preferences (Mulder et al. 2009). The body length was measured to the nearest 1 µm with an eyepiece micrometer, and life stage and gender were recorded as well (all data downloadable from *Ecological Archives*: Mulder and Vonk 2011). Soil nematodes exhibit on average body lengths ∼500 µm, ranging from 0.1 mm to 1.3 mm for Dauerlarvae and up to 5 mm for adults. Computations used SAS version 9.1.3 Service Pack 3, SPSS 16.0, ACCESS XP, and EXCEL 2007, and the Visual Basic optimization toolbox.

## Results

**Figure 1:**
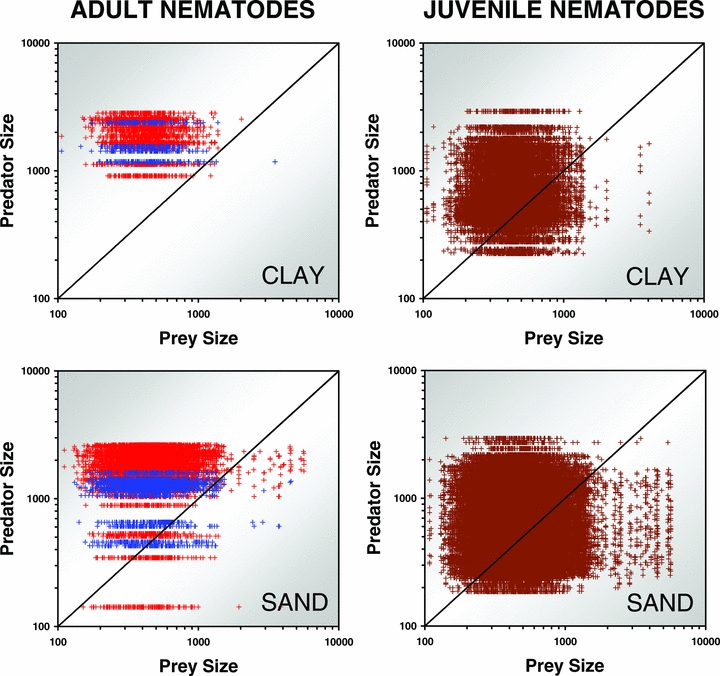
Predator–prey body size relationships for soil nematodes in managed grasslands on clay (upper panels) and sand (lower panels). The body lengths (µm) of all possible targets (males, females, and juveniles at all developmental stages) were plotted against the occurring predators either as adults (left panels, male consumers in blue and female consumers in red) or as juveniles (right panels, in brown). The diagonal 1:1 lines show equal body size for both prey and predatory nematodes. About 6.3% of the nematodes recorded in clay and 7.3% in sand belong to the second trophic level (obligate and facultative predatory nematofauna). The coefficients of variation for all predator–prey interactions in adult and juvenile consumers are 49% and 69% in clay, and 54% and 78% in sand, respectively.

The results show striking dichotomies between the two predatory life stages, that is, juvenile versus adult nematodes (Fig. 1). As a matter of fact, much broader feeding strategies (implying potential aggressive behavior) can be recognized for juvenile consumers. In contrast to adult consumers, juveniles might attack more often larger targets (32.11% of the predatory juveniles share a larger potential prey). Many juvenile predators are smaller than their potential prey, like the widely occurring Neodiplogasteridae (their average predator–prey body size ratio in clay equals 0.92 ± 0.55 SD and in sand 0.84 ± 0.49 SD). Each veil line running parallel to the prey size axis represents one individual predator, in most cases one single adult (Fig. 1, left panels).

According to the loamy or sandy structure of the soil systems, predatory adults share significantly different body size ratios between the nematofauna in clay and in sand (ANOVA *P* < 0.0001: *F_♂_* = 331 and *F_♀_* = 101). In clay, only 0.30% of the targeted prey exceeds the length of the predatory adults; in sand, 3.89% of the prey exceeds the length of the predatory adults. Such a mechanism resulted in skewed predator–prey distributions according to the predatory life stage and gender: the average predator–prey body size ratios for females, males, and juveniles are 4.94, 3.97, and 1.89 in clay, and 4.47, 2.77, and 1.75 in sand, respectively. If we compute the same average ratios for juveniles with either stylet or large buccal cavities, the predator–prey body size ratios become 1.89 in clay and 2.21 in sand, and 1.80 in clay and 1.05 in sand, respectively.

At each location, all developmental stages were recorded: on the average, the total adult-to-juvenile ratio of nematodes was 0.31 (± 0.10 SD). As each taxon was not always present with both genders on the same moment at the same site, intraspecific temporal variation of the female-to-male ratio was inflated and the total female-to-male ratio of adult nematodes (averaging 3.48 ± 2.78 SD) did not show variation through time. In contrast to adults, juveniles show a remarkable difference in their predator–prey body size ratios according to their feeding behavior and the soil microenvironment. In particular, predatory juveniles with large buccal cavities seem to be much more aggressive in coarse sandy soils than in clay, potentially attacking prey as large as themselves (their average predator–prey body size ratio equals 1.05 ± 0.74 SD). Neodiplogasteridae, with their esophageal sucking attacks, and the stylet-bearing *Dorylaimoides* and *Aporcelaimellus*, are common predatory juveniles. (*Aporcelaimellus* is also frequent as predatory adult, mostly as female.) In sand, stylet-bearing predators are about twice as frequent as those with wide buccal cavities; in clay, where Neodiplogasteridae are rare, stylet-bearing predators are even four times more frequent than other predators.

Taxon-related body size averages (± SD) were shown in Table 1: at higher trophic levels (bold taxa) Mononchidae, Qudsianematidae, and Thornenematidae are the most widespread predatory nematodes. *Anatonchus, Clarkus, Mylonchulus, Seinura*, and *Tripyla* are stenophagous predators, the other families and genera given in Table 1 as bold belong to euryphagous predators, comprehending all the not obligate predators. One example for “non obligate predation” is provided by *Pungentus*. Originally described as plant-feeding nematode (Thorne and Swanger 1936; Trudgill 1976), this genus was recognized by Yeates et al. (1993) to have not only “plant ectoparasitism” as preferred feeding habit, but to occur as predatory nematode as well (see Small 1987, his Table 3, and Ferris and Ferris 1989, their Table 2), being “omnivore.”

**Table 1:** Nematode length in µm (mean ± SD) of juveniles, females, and males for widespread taxa, that is, preys recorded more than 100 times (in regular font) and predators recorded more than five times (in bold). We summarized here 95% of the total of 15,522 individuals (single observations in brackets). We performed Kruskal–Wallis ANOVA for testing the null hypotheses of either no difference in the body size between the soil types (coarse sand vs. loamy clay) or between the life stages (juvenile vs. adults). The developmental growth was clearly recognizable; juvenile Dauerlarvae were per se excluded from the latter Kruskal–Wallis analysis, but the closely correlated Rhabditidae exhibit a sharp difference in length between their juvenile and adult stages (*P* < 0.001)

	Sand			Clay		
		
Taxon	Juvenile	Female	Male	Juvenile	Female	Male
*Acrobeles*	378 ± 101	569 ± 118	528 ± 142			
*Acrobeloides*	342 ± 103	456 ± 109	(529)	310 ± 71	489 ± 127	
*Aglenchus*	446 ± 81	523 ± 70	520 ± 50	391 ± 61	488 ± 91	492 ± 86
*Anaplectus*	566 ± 245	1008 ± 153	848 ± 303	491 ± 245	1052 ± 206	1202 ± 253
***Anatonchus***	**613 ± 378**			**954 ± 389**	**(2167)**	**(2386)**
*Aphelenchoides*	341 ± 108	593 ± 216	534 ± 116	348 ± 138	599 ± 157	546 ± 91
***Aporcelaimellus***	**1204 ± 492**	**2152 ± 300**	**(1428)**	**1215 ± 571**	**2253 ± 493**	
*Bitylenchus*	874 ± 345	956 ± 312	694 ± 135		715 ± 146	737 ± 128
***Clarkus***	**742 ± 267**	**1095 ± 294**		**534 ± 225**		
Dauerlarvae	480 ± 163			476 ± 138		
Dolichodoridae	370 ± 130	(637)	539 ± 12	481 ± 212		(419)
***Dorylaimoides***	**721 ± 334**	**1158 ± 2**	**1218 ± 79**	**590 ± 314**	**1415 ± 710**	**(1550)**
***Epidorylaimus***	**1141 ± 382**	**1558 ± 168**				
*Eucephalobus*	353 ± 95	551 ± 92	516 ± 94	347 ± 94	553 ± 81	537 ± 85
***Eudorylaimus***	**1054 ± 683**	**1736 ± 453**	**(1232)**	**965 ± 447**	**1778 ± 111**	
*Filenchus*	462 ± 116	559 ± 128	514 ± 118	445 ± 115	539 ± 85	532 ± 56
*Helicotylenchus*	502 ± 140	770 ± 99		476 ± 140	686 ± 80	
*Heterodera*	502 ± 58			488 ± 49		
*Meloidogyne*	408 ± 30			424 ± 31		
***Mesodorylaimus***	**1026 ± 236**	**1457 ± 588**	**1380 ± 212**	**(1509)**		
**Mononchidae** undiff.	**484 ± 166**			**1051 ± 446**		
***Mononchus***	**623 ± 248**	**(2243)**		**636 ± 239**		
***Mylonchulus***	**698 ± 345**			**(473)**		
**Neodiplogasteridae**	**354 ± 131**	**462 ± 102**	**564 ± 120**	**387 ± 190**		
*Panagrolaimus*	441 ± 131	789 ± 132	721 ± 122	479 ± 158	829 ± 117	733 ± 107
*Paratylenchus*	324 ± 44	347 ± 41	354 ± 33	296 ± 52	350 ± 44	343 ± 6
*Plectus*	511 ± 277	649 ± 206	966 ± 55	616 ± 362	944 ± 607	
*Pratylenchus*	293 ± 78	487 ± 67	420 ± 39	290 ± 79	504 ± 59	477 ± 77
***Prodorylaimus***	**1005 ± 230**	**(1539)**		**1077 ± 148**		
***Pungentus***	**1321 ± 324**	**1663 ± 179**		**842 ± 439**	**(1651)**	
**Qudsianematidae** undiff.	**881 ± 357**	**1299 ± 127**		**523 ± 176**	**(1674)**	
Rhabditidae	437 ± 161	787 ± 225	633 ± 156	417 ± 146	833 ± 216	720 ± 120
***Seinura***	**388 ± 46**		**(458)**	**(416)**		
***Thonus***	**(1290)**		**1265 ± 142**		**(2520)**	
**Thornenematidae** undiff.	**823 ± 323**	**(1743)**	**1356 ± 86**	**788 ± 351**		
***Tripyla***	**766 ± 280**	**1395 ± 157**	**1276 ± 67**	**726 ± 293**	**1433 ± 272**	**1260 ± 144**
Tylenchidae	378 ± 101	507 ± 163	528 ± 121	377 ± 110	523 ± 144	504 ± 104
*Tylenchorhynchus*	497 ± 202	768 ± 230	600 ± 102	411 ± 144	615 ± 363	

## Discussion

Nematode body size measurements were defined at the individual level using one comprehensive and unbiased sampling method, so that the length distribution incorporates both the intraspecific variation and the soil nematofauna composition. In the latter case, the nematode life cycles clearly show different survival and dispersal strategies (Small 1987; Yeates 1987; Bongers 1988). Although we were unable to address all possible effects of seasonality and temperature on the nematofauna composition (cf. Yeates 1968; Ritz and Trudgill 1999; Mulder et al. 2003), the correlations between sampling time and total adult-to-juvenile ratio in all pooled sites—and in all sites belonging to one soil type separately—were in our case not significant (*P* > 0.14).

Predatory adults are on average three times up to five times as large as their prey, whereas juveniles seem to search their prey randomly and without preference, possibly abandoning their prey before fully utilizing it or dealing the ingestion of their prey as group activity. For example, *Seinura* juveniles might attack Rhabditidae, *Eucephalobus*, Tylenchidae, and *Paratylenchus*, injecting toxic esophageal secretions into the injured prey (Hechler 1963; Bilgrami 2008) and often feeding together with other predatory nematodes. Stylet-bearing nematodes (predators without wide oral apertures) cannot swallow their prey and are forced to digest parts of their prey outside their esophagus because their oral apertures are too narrow for a direct ingestion (Bilgrami 2008). This implies that predatory nematodes with wide oral apertures such as mononchs (Cobb 1917; Andrássy 1958) are supposed to be more sensitive to the size of their prey than other predators such as diplogasterids that can either (1) feed on prey much larger than themselves thanks to extracorporal digestion or (2) suck the body content of prey leaving an empty cuticle behind them.

But mononchs also need one lip contact to detect their prey (Grootaert and Maertens 1976; Grootaert and Wyss 1979). To compensate the short range of their prey detection, mononchs such as *Mylonchulus* are actively mobile (Grootaert and Maertens 1976; Bilgrami 1992), a mechanism that contributes to explain their higher sensitivity: a prey encounter is enhanced by an efficient searching ability and rapid attacks (Grootaert and Wyss 1979), and the chance to hold and suck a large prey becomes greater in the case of a larger sized target.

The relatively high degree of large prey attacked by juveniles can be explained by either enzymatic injection during extracorporal digestion, as known for other invertebrates such as arthropods (Cohen 1995), although some predatory juveniles during the first two development stages survive in cultures on agar (Small 1987; Yeates 1987; but see also Linford and Oliveira 1937). Moreover, soil predatory nematodes are known to attack randomly other worms much larger than themselves, such as enchytraeids (Small 1987; Mulder and Elser 2009), as soon targeted (enchytraeid) worms share either low “behavioral resistance” (less undulation) or low “physical resistance” (no thick cuticles). Wounding and prey vulnerability (sensu Bilgrami 1992) are major challenges in soil nematology because the entire scale of possible predator–prey interactions can be strongly enhanced from those presented in this study. Attraction and aggregation of soil predatory nematodes around the targeted resource, suggesting possibly importance of (1) stimulating prey secretions in establishing predator–prey contacts and (2) size and numerical abundance of preys in determining patches of predators, were recognized by Bilgrami and Gaugler (2004).

## Conclusions

Given the need to discriminate between the fundamental trade-offs (habitat-response relationships at organism level) and the secondary trade-offs (habitat-response relationships at species level), gender and life stage have to be taken into account in multitrophic networks. The incorporation of nematodes into feeding habits according to their buccal size and their buccal armature is common practice (Wieser 1953; Yeates et al. 1993) and defines the likelihood of behavioral feedings (Wieser 1953; Yeates 1987; Bilgrami and Gaugler 2004). In our nematode predator–prey systems, the measurable variation in attack rates with corresponding predator–prey oscillations seems to depend not only on taxonomy, but on gender and life stage as well. Gender itself might contribute to explain a possibly large part of the behavior, a conclusion partially supported by recent simulations on how the growth rate and the phenology are influenced by the size variability present through time (Moya–Laraño 2011). Although simplistic conclusions must be avoided, we measured the variation of predator–prey body size ratios in soils; showed that the nematode life stage strongly influences the size range of possible targets; assessed that males seem to be more aggressive than females in their feeding behavior (yielding contrasting prey's responses); and argued the physiological validity of using species-specific body size average as sole independent predictor in allometric models. These results provide evidence that gender and life stage clearly matter at different levels in community ecology.
